# Investigation on the origin of hot electrons in laser plasma interaction at shock ignition intensities

**DOI:** 10.1038/s41598-023-46189-7

**Published:** 2023-11-24

**Authors:** G. Cristoforetti, F. Baffigi, D. Batani, R. Dudzak, R. Fedosejevs, E. D. Filippov, P. Gajdos, L. Juha, M. Khan, P. Koester, M. Krus, D. Mancelli, A. S. Martynenko, Ph. Nicolai, S. A. Pikuz, O. Renner, A. Tentori, L. Volpe, N. Woolsey, G. Zeraouli, L. A. Gizzi

**Affiliations:** 1 Istituto Nazionale di Ottica, CNR, Pisa, Italy; 2 Université de Bordeaux, CNRS, CEA, CELIA, 33405 Talence, France; 3 Institute of Plasma Physics of the CAS, Prague, Czech Republic; 4 Institute of Physics of the CAS, Prague, Czech Republic; 5 University of Alberta, Edmonton, Canada; 6 JIHT RAS, Moscow, 125412 Russia; 7 York Plasma Institute, School of Physics, Engineering and Technology, University of York, York, UK; 8 Institute of Plasma Physics and Lasers, Hellenic Mediterranean University Research Centre, Rethymnon, Greece; 9 Department of Electronic Engineering, Hellenic Mediterranean University, Chania, Greece; 10 GSI Helmholtzzentrum für Schwerionenforschung, Darmstadt, Germany; 11 NRNU MEPhI, Moscow, 115409 Russia; 12 The Extreme Light Infrastructure ERIC, Dolni Brezany, Czech Republic; 13 Centro de Laseres Pulsados (CLPU), 37185 Villamayor, Salamanca Spain; 14 ETSI Aeronáutica y del Espacio, Universidad Politécnica de Madrid, 28040 Madrid, Spain

**Keywords:** Plasma physics, Laser-produced plasmas

## Abstract

Shock Ignition is a two-step scheme to reach Inertial Confinement Fusion, where the precompressed fuel capsule is ignited by a strong shock driven by a laser pulse at an intensity in the order of $10^{16}$ W/cm$^2$. In this report we describe the results of an experiment carried out at PALS laser facility designed to investigate the origin of hot electrons in laser-plasma interaction at intensities and plasma temperatures expected for Shock Ignition. A detailed time- and spectrally-resolved characterization of Stimulated Raman Scattering and Two Plasmon Decay instabilities, as well as of the generated hot electrons, suggest that Stimulated Raman Scattering is the dominant source of hot electrons via the damping of daughter plasma waves. The temperature dependence of laser plasma instabilities was also investigated, enabled by the use of different ablator materials, suggesting that Two Plasmon Decay is damped at earlier times for higher plasma temperatures, accompanied by an earlier ignition of SRS. The identification of the predominant hot electron source and the effect of plasma temperature on laser plasma interaction, here investigated, are extremely useful for developing the mitigation strategies for reducing the impact of hot electrons on the fuel ignition.

## Introduction

**Figure 1:**
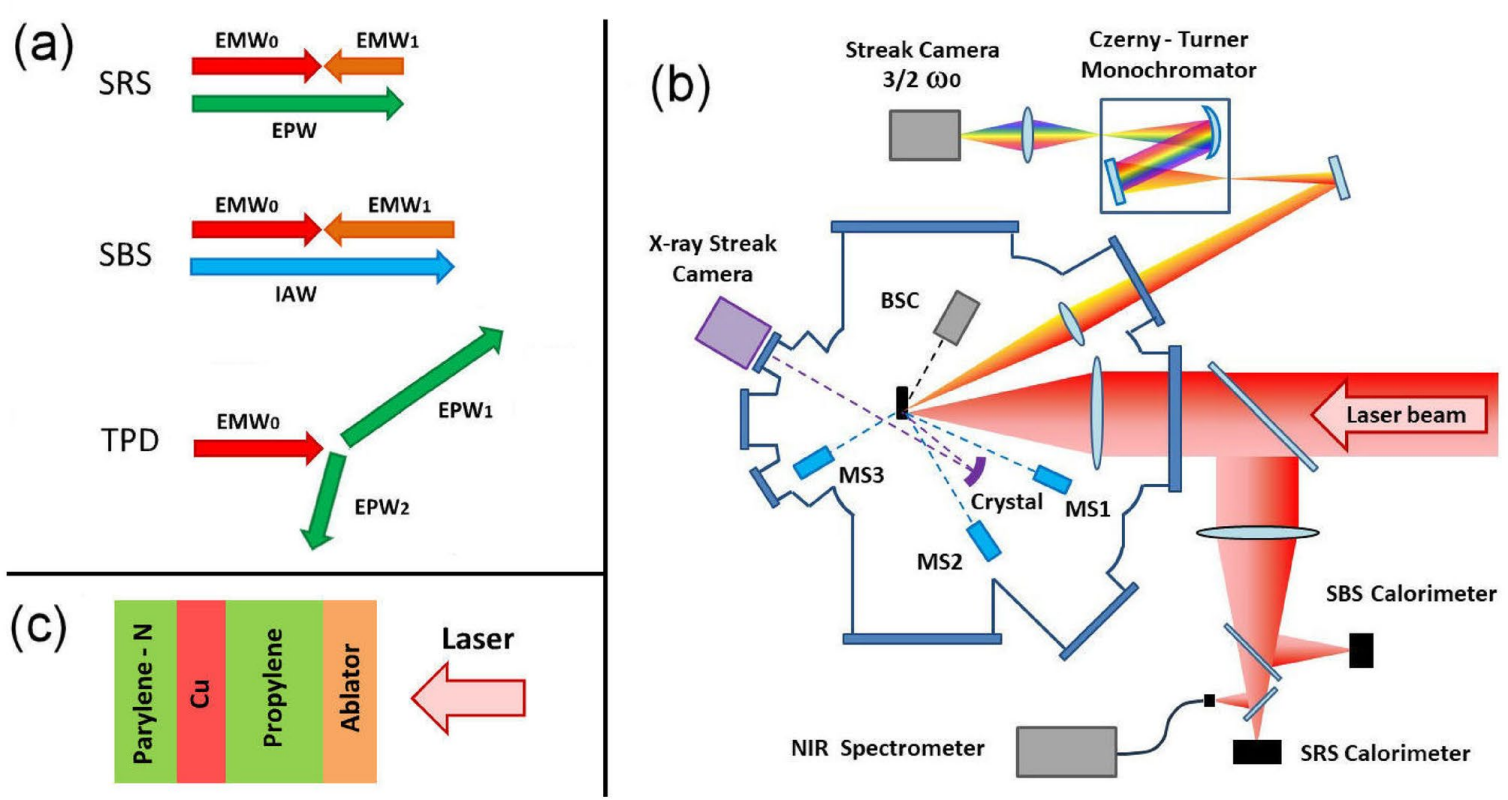
(**a**) Schematic diagram of backward Stimulated Raman Scattering (SRS), backward Stimulated Brillouin Scattering (SBS) and Two Plasmon Decay (TPD), where the arrows represent the wavevectors of laser light (EMW$_0$), scattered light (EMW$_1$), electron plasma waves (EPW, EPW$_{1,2}$) and ionic acoustic wave (IAW). (**b**) Scheme of the experimental setup. The acronyms BSC and MS1, MS2 and MS3 indicate the Bremsstrahlung cannon spectrometer and the three electron magnetic spectrometers. (**c**) Target multilayer structure, where the ablator can be Parylene-N coated by 40 nm aluminium, carbon, aluminium, titanium or nickel.

The interaction of a nanosecond laser pulse with a solid target at intensities of $10^{16}$ W/cm$^2$ is still poorly known and a challenge to model, since it belongs to a transition region between different mechanisms of absorption of the laser pulse. While at laser intensities in the range ($10^{13}-10^{15}$) W/cm$^2$ the collisional absorption of the laser light (i.e. via inverse Bremsstrahlung process) is in fact predominant, here collisions become less effective since the quivering velocity of the electron in the laser field becomes comparable to the thermal velocity^1^. On the other hand, non collisional absorption processes involving the excitation of collective ion or electron plasma waves begin to be quantitatively important and to exhibit a non linear behaviour^2^. The inelastic scattering of the laser light with density fluctuations can in fact drive the excitation of electron or ion acoustic plasma waves (Fig. 1a)—via the Stimulated Raman Scattering (SRS) and the Stimulated Brillouin Scattering (SBS)^2,3^—resulting in a considerable conversion of incident laser light to redshifted light with frequency $\omega _0-\omega _e$ and $\omega _0-\omega _i$, where $\omega _0$, $\omega _e$ and $\omega _i$ are the laser, electron and ion-acoustic wave frequencies, respectively. This light is diverted out of the plasma and therefore consists in a net loss of laser energy. In addition, laser light can generate plasma waves with frequency $\approx \omega _0/2$ in the proximity of a quarter of the critical density $n_c$ of the laser light, where $n_c=m\omega _0^2/4\pi e^2$ is the electron density at which the wavevector *k* of the laser light vanishes and the laser is reflected. At densities $\approx n_c/4$, in fact, the Two Plasmon Decay (TPD)^4^ instability can be driven (Fig. 1a), for which laser photons decay into pairs of electron plasma waves, one moving forward and the other backward with respect to the density gradient, with energy $\omega _{e1} = \omega _0/2 + \Delta \omega (n_e,T)$ and $\omega _{e2} = \omega _0/2 - \Delta \omega (n_e,T)$, respectively, where $\Delta \omega (n_e,T)$ is a small correction depending on the values of density and temperature. The longitudinal electric field of electron plasma waves driven via SRS or TPD can in turn transfer energy to a subset of electrons, called ‘hot’ electrons (HE), reaching suprathermal energies of tens to hundreds of keV, allowing them to escape the plasma and penetrate the solid target.

A reliable description of the above parametric instabilities is a key issue in inertial confinement fusion^5,6^, since both the light scattered from the plasma and the hot electrons can account for several tens of percent of incident laser energy, therefore affecting significantly the evolution and the energy balance of plasma hydrodynamics and producing a larger energy requirement for the laser driver. Moreover, these mechanisms may exhibit a collective behaviour through the concomitant action of several overlapping laser beams^7–9^, which can affect the energy balance of the different beams and result in an asymmetric compression of the fuel capsule. Furthermore, in direct-drive schemes^10^ for inertial confinement fusion, HE can preheat the uncompressed fuel, enhancing its entropy and preventing its ignition. The impact of parametric instabilities is particularly critical in the direct-drive Shock Ignition scheme^11^, where the fuel ignition is triggered by a strong shock wave launched by a laser spike at an intensity of $\sim 10^{16}$ W/cm$^2$ impinging on a precompressed capsule surrounded by a mm-scale plasma corona. The laser intensity envisaged for SI is an order of magnitude higher than in conventional direct-drive schemes, which dramatically increases the extent of parametric instabilities, their non linear character, and the importance of kinetic effects on their evolution. In this scheme, the role of HE is also not fully understood, since they are generated when the fuel capsule is already strongly compressed with a shell areal density of $\langle \rho r\rangle {\approx } (50-80)$ g/cm$^2$, so that the range of HE can be smaller or larger than the thickness of the compressed shell, depending on their energy^12^. According to recent works^13,14^, low energy HE of few tens of keV could be stopped in the compressed shell, with the beneficial effect of reinforcing the ignitor shock, while HE with energy higher than ${\approx } 100$ keV could cross the shell and preheat the fuel.

This situation therefore calls for an accurate investigation of the energy distribution of HE in Shock Ignition conditions and of the mechanisms of their generation, in order to figure out their impact on the fuel ignition and to setup strategies for its mitigation. As mentioned above, SRS and TPD are expected to be major sources of HE in Shock Ignition conditions, even if additional processes, as for example resonance absorption or plasma cavitation, could also contribute to generate HE. Both SRS and TPD can grow with a convective or absolute character^15^, depending on the values of density and density gradients in the region where they are driven; this detail determines the energy distribution of the generated HE. The prevalence of SRS or TPD also strongly affects the energy of HE, and therefore their impact on the implosion performance.

Recent experiments carried out at the Vulcan^16^, OMEGA^17^ and LMJ^18,19^ laser facilities, in conditions of laser intensity in the order of $10^{16}$ and long plasma scalelength ($L_n = n/(dn/dx) =$ 300–500 $\upmu$m), showed the growth of convective SRS at relatively low plasma densities $n\approx$ 0.05–0.10 $n_c$, likely driven in plasma filaments, and recorded no signatures of TPD. All these experiments therefore suggest that SRS could be the main source of HE and that TPD tends to disappear at laser intensities and density scalelength plasmas envisaged for the Shock Ignition scheme. However, the dependence of SRS and TPD competition on plasma temperature, and its consequences on HE generation still requires investigation for a more complete understanding of laser-plasma interaction at Shock Ignition laser intensities^15,20,21^.

Here, we report the results of an experiment carried out at laser intensity in the range (0.5–1.0)$\cdot 10^{16}$ W/cm$^2$, where the diagnostics were designed to characterize in detail the spectra, the time history and the energy extent of hot electrons and SRS/TPD instabilities. The aim of the experiment was twofold: (a) establish the process responsible for the HE generation and (b) investigate the dependence of the competition between TPD and SRS on plasma temperature. For this second purpose, we varied the plasma temperature in the range $3<T_{keV}^{max}<5.4$ by using ablators of different composition.

## Experimental set-up

### Interaction conditions

The experiment was performed in planar geometry at the Prague Asterix Laser System (PALS) facility, by using a single laser pulse responsible for both creating the plasma and driving the instabilities. A scheme of the experimental setup is shown in Fig. 1b. The laser pulse ($\lambda _{0}$ = 1314 nm, 300 ps, 700 J) was smoothed by a random phase plate and focussed at normal incidence on a flat target to a $\approx$ 100 $\upmu$m (FWHM) Gaussian focal spot. The f/$\#$-number of the focusing lens, corresponding to the ratio between the focal length and the effective diameter of the lens, had a value f/$\#$2. Considering the amount of energy enclosed in the spot, accurately determined by dedicated calorimetric measurements, the peak laser intensity on the target surface was varied in the range $I=$ (0.5–1.0) $\cdot 10^{16}$ W/cm$^{2}$. The targets consisted of thin multilayer foils (Fig. 1c), including (a) a 20 $\upmu$m-thick ablation layer, determining laser-plasma interaction and the generation of hot electrons, followed by (b) a 50 $\upmu$m-thick propylene layer (C3H6, Z$_{av}$ = 5.52), where forward-accelerated HE could propagate, (c) a 10 $\upmu$m-thick copper layer, used as a tracer of HE via K$_{\alpha }$ line emission, and finally (d) a 20 $\upmu$m-thick parylene-N layer (C16H16, Z$_{av}$ = 5.61), with the role of reducing the effect of HE refluxing on the Cu K$_{\alpha }$ emission. Different ablation materials, including parylene-N, carbon (Z = 6), aluminum (Z = 13), titanium (Z = 22) and nickel (Z = 28), were used, with the aim of modifying the interaction conditions and, in particular, the temperature of the plasma, expected to rise with the Z-number of the material. The parylene-N transparent layer was also coated by a 40 nm Al flash, in order to avoid the propagation of the laser into the target at early times of interaction. Radiative-hydrodynamic simulations have been carried out by using the CHIC code^22^ to determine the plasma parameters along the laser pulse interaction and to favour a correct interpretation of the experimental data. Simulations show that the plasma temperature in the underdense region reaches a maximum value $T_{max}$ in proximity of the laser peak for all the targets, with $T_{max}$ rising monotonically with the Z-number of the ablator material and passing from $T_{max}=3.3$ keV for Parylene-N to $T_{max}=5.4$ keV for the Ni. The density scalelength $L_n$ of the plasma in the underdense plasma monotonically rises during laser irradiation showing only small variations with the ablator composition, which begin to be appreciable only at late times of interaction, e.g. $(\Delta L_n / L_n)^{max}\approx 0.15$ at 200 ps after the laser peak. $L_n$ value at the densities of interest for SRS and TPD $n =$ 0.10–0.25 $n_c$ is 90–100 $\upmu$m at laser peak time for all the ablators.

### Diagnostics

Diagnostics were chosen with the aim of characterizing quantitatively, spectrally and temporally the parametric instabilities, through the analysis of scattered light, and the generation of hot electrons, via X-ray spectroscopy and HE magnetic spectroscopy.

The light backscattered in the focussing cone was split in different channels for spectral and quantitative characterization by means of time-integrated infrared spectroscopy and calorimetric techniques. Spectral analysis of light was made possible by an NIR spectrometer NIRQuest Ocean Optics coupled to an IR low-OH optical fiber, with a spectral range of 1100–2530 nm, thus including the light backscattered by convective SRS but excluding the $\omega _{0}/2$ light at $\lambda$ = 2628 nm and the longer wavelengths. The amount of light backscattered by SRS and by SBS was obtained by means of two dedicated calorimeters with suitable optical filters; values of SRS and SBS reflectivity could then be calculated after the calibration of the spectral transmission of the optical line in the infrared range. It is worth stressing that SBS reflectivity indeed includes any contribution from elastic (non SBS) laser light scattering since the bandpass filter at $\lambda \approx 1314$ nm (FWHM = 10 nm) was not able to discriminate between the two components. Light scattered at $30^{\circ }$ in the horizontal plane was also spectrally resolved by a Czerny–Turner monochromator with 150 l/mm or 300 l/mm gratings and successively time-resolved by a Hamamatsu C7700 optical Streak Camera at a maximum temporal resolution of 15 ps. This setup allowed a detailed characterization of ${\frac{3}{2}} \omega _{0}$ harmonics, including contributions of both SRS and TPD instabilities. In the configuration with the lower spectral resolution, i.e. with the 150 l/mm grating, a frequency-doubled pickoff of the laser beam was used as time fiducial for absolute temporal calibration of laser-plasma interaction with respect to the laser pulse.

$K_{\alpha }$ emission of copper ($\lambda = 1.5406$ Å) was produced by the collisions of HE with the Cu tracer layer, resulting in the $2p\rightarrow 1s$ K-shell fluorescence. Time- and space-resolved measurements of the $K_{\alpha }$ line were carried out by imaging the Cu emission on a Hamamatsu high dynamic range X-ray streak camera by means of a spherically bent crystal of quartz (422). The signal, acquired from the front side of the target at $33^{\circ }$ from the normal, covered a spectral window $\Delta \lambda = 1.4$ mÅ which was sufficient to fully include the Cu $K_{\alpha 1}$ emission from the cold target. Absolute time calibration with respect to the laser pulse was made possible by sending a pickoff of the laser beam, converted in third harmonics, onto the slit of the X-ray streak camera.

A Bremsstrahlung Cannon Spectrometer (BSC)^23^ using K-edge and differential filtering (14 filters of increasing Z from Al to Pb) was located at a distance of 220 mm from the target in the horizontal plane on the front side and measured the X-ray spectrum at $61^{\circ }$ from the normal to the target; the signal, detected by Imaging Plates, was mainly produced by the Bremsstrahlung emission of HE propagating into the target, and could therefore be utilized to indirectly calculate the temperature of the hot electron distribution.

Finally, three HE Magnetic Spectrometers (MS), equipped with a 1 mm wide collimator and 80 mT magnets, were located at 300 mm from the target, two looking at the front side at $25^{\circ }$ and $51^{\circ }$ and one at the rear side at $31^{\circ }$. They recorded the spectrum of electrons in the range 0.1–2 MeV through the energy-spatial dispersion across imaging plate detectors.

## Results and discussion

### Hot electrons

**Figure 2:**
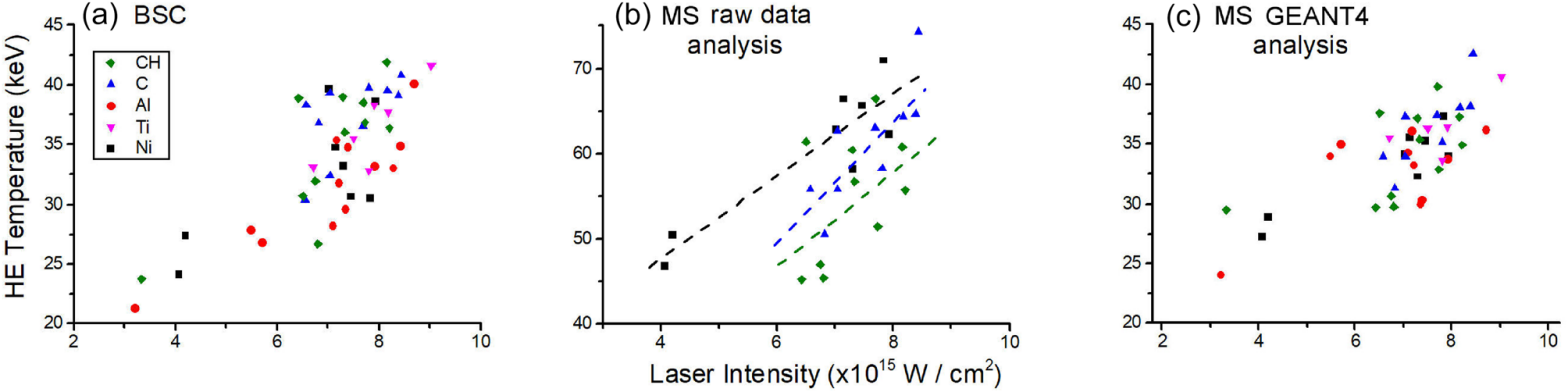
(**a**) Temperature of HE propagating into the target calculated from a) BSC and (**b**,**c**) MS. The values in (**b**) have been obtained by fitting the energy spectra extracted from the raw data while the values in (**c**) have been corrected for the effects of electron transport through the target. The dashed lines in graph (**b**) are guides for the eye to show the dependence on the ablator material. The uncertainty, not shown, is (10–20)% depending on the shot.

A detailed analysis of data obtained by the BSC, the MS, and the K$_{\alpha }$ imaging spectrometer can be found in a companion paper^24^, together with a critical discussion of diagnostics for a correct characterization of HE. Here, we report the main results, needed for the aim of the present paper, i.e. investigating the origin of the HE.

Energy and number of HEs propagating into the target were obtained from the measurements of Bremsstrahlung cannon and of the MS located beyond the target. All the Imaging Plates located in the diagnostics were scanned after approximately 20 min from exposure. In the BSC only the first 6 Imaging Plates recorded a usable signal, where the first one was typically ignored from further analysis due to its susceptibility to the plasma self-emission. For the remaining Imaging Plates, the signal was obtained by extracting the photostimulated luminescence from a region of interest and by subtracting the background measurement taken from an unexposed Imaging Plate. The HE energy distribution was found by comparing the extracted data with synthetically generated signals by a residual sum analysis. Synthetic signals were generated using Geant4 Monte-Carlo simulations^25^, accounting for both the Bremsstrahlung emission from hot electrons that were injected into a target, and for the response of the detector to incident radiation. It should be noted that Geant4 code is not able to model the effects of the plasma on the HE propagation and does not account for the target expansion; however, these effects have been previously estimated to be negligible^26^. The temperature, $T_{hot}$, and total population number, $N_{HE}$, were found by assuming for the synthetic signals that the HE had a 3D Maxwell-Boltzmann distribution $f(E,T_{hot})= (2/\pi ^{1/2}T_{hot}^{3/2})E^{1/2}\exp (-E/T_{hot})$. A full description of the procedure is outlined in Refs.^16,27^. As shown in Fig. 2a, the temperature of HE rises from 25 to 40 keV when laser intensity increases from $3\cdot 10^{15}$ W/cm$^2$ to $9\cdot 10^{15}$ W/cm$^2$, without any marked dependence on the ablator composition. The conversion efficiency from laser to HE energy is in the range (0.6–2.0%).

The HE temperature could be also calculated from electrons emerging from the rear side of the target and measured by the magnetic spectrometer. Electron spectra, shown in Ref.^24^, exhibited an exponential decay behaviour in their high energy tail ($E>100$ keV); HE temperature was therefore calculated by fitting this region of energy with a Maxwellian function of the same form as described above. As shown in Fig. 2b for the CH, C and Al multilayer targets, the values obtained ranged from 45 to 70 keV, with an appreciable dependence on the composition of the ablator material. Intensive Geant4 simulations were therefore carried out to investigate the effects of electron transport through the target on the energetic spectrum of the emerging electrons. These simulations did not include the impact of the plasma nor the rear surface sheath field on the electron propagation. The procedure allowed to correct the dependence of the measured energetic spectra on the composition of the target and to estimate the temperature of a Maxwellian population of HE injected into the target on the front side. Results made evident that the ablator dependence of the HE temperatures, visible in Fig. 2b, was produced by HE propagation through the target, and particularly through the ablator material. As shown in Fig. 2c, the temperatures of generated HEs, estimated by this procedure, were all in the range 25-40 keV without any marked dependence on the ablation material, well in agreement with those calculated from the BSC.

Time-resolved imaging of Cu $K_{\alpha }$ emission was here used to infer the time history of HE generation. An essential issue was distinguishing the contribution of Cu $K_{\alpha }$ emission from Bremsstrahlung emission produced by HE propagating into the target and X-ray continuum emission from the plasma corona, in the total signal acquired from the X-ray imaging spectrometer. To estimate this we carried out GEANT4 simulations for all the targets to compare the $K_{\alpha }$ photons emitted per steradian in the Cu tracer layer and the photons produced by Bremsstrahlung collisions throughout the target. We injected a Maxwellian distribution of HE, with values of temperature and absolute population determined by the BSC measurements. The amount of X-ray photons emitted from the plasma corona in front of the target, due to Bremsstrahlung and recombination processes, was also estimated by postprocessing the plasma density profiles obtained by CHIC hydrodynamic simulations with the collisional-radiative FLYCHK code. The total number of photons was estimated by integrating over a 300 ps time window, centered on the laser peak. The comparison of the X-ray photon numbers emitted in the spectral window covered by the imaging system ($\Delta \lambda = 1.4$ mÅ) revealed that Cu $K_{\alpha }$ emission largely dominated on the continuum emission for parylene-N and carbon ablators, while corona plasma emission overwhelmed the Cu $K_{\alpha }$ emission approximately by 1 order of magnitude for Ti and Ni targets. In case of aluminium ablator, simulations predict that $K_{\alpha }$ emission exceeds the background continuum emission by only a factor 2–3. Measurements performed with a time integrating high resolution x-ray spectrometer, reported in Ref.^24^, confirmed the results of the simulations, showing that the targets with Ti and Ni ablators cannot be used for time resolved Cu $K_{\alpha }$ imaging, whereas the signals collected with Parylene-N, C and Al multilayer targets (achieving approximately one half of the signal measured on CH and C coated targets) provide reliable data. Here we focus on time-resolved Cu $K_{\alpha }$ measurements from Parylene-N and carbon multilayer targets to infer the timing of HE generation; selected results of time resolved measurements with Al targets can be found in Ref.^24^

**Figure 3:**
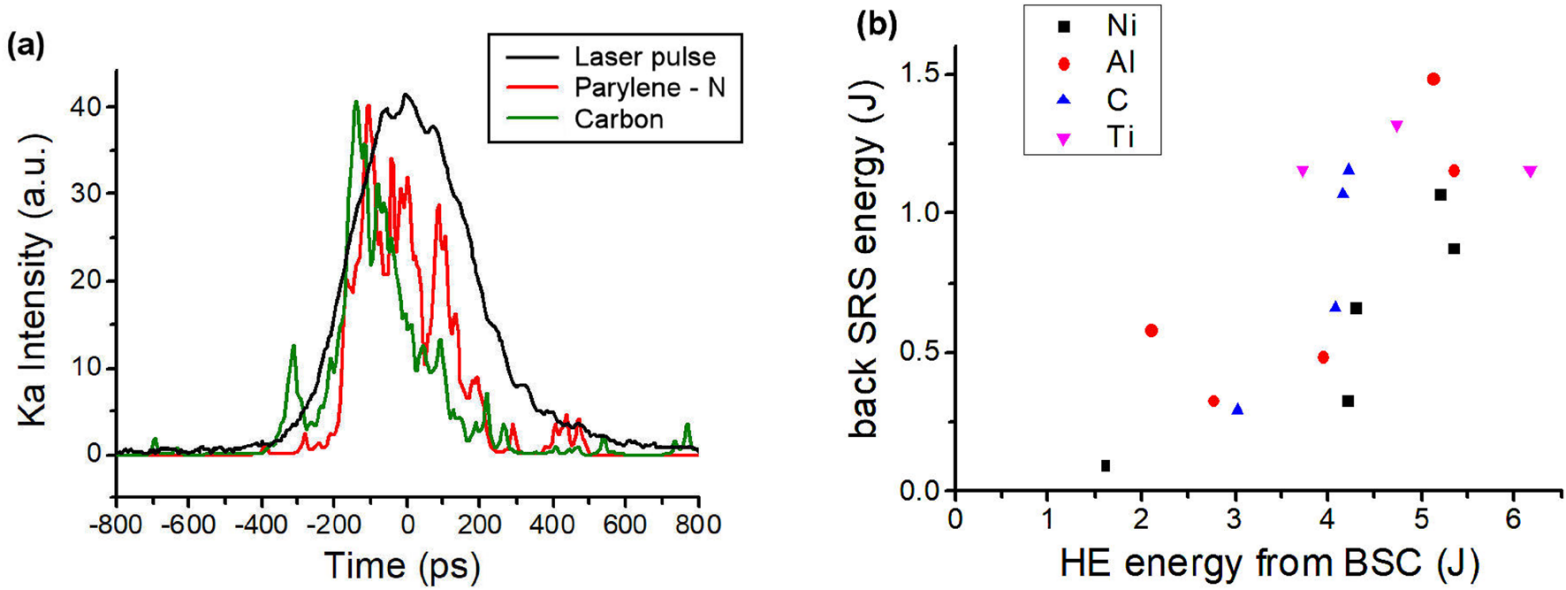
(**a**) temporal profile of Cu $K_{\alpha }$ emission and laser pulse for shots over parylene-N and carbon multilayer targets. The relative intensity of the different curves is arbitrary. (**b**) correlation between the energy of backscattered SRS, measured by the calorimeter, and the energy of HE, obtained by Geant4 analysis of BSC data. Only homogeneous data acquired with the same experimental setup have been included, which explains the lack of Parylene-N shots in this graph.

X-ray streak data show that the duration of Cu $K_{\alpha }$ emission (*FWHM* = 160–300 ps) is typically shorter than the laser pulse duration (*FWHM* = 310–360 ps) and peaked 50–100 ps before the laser maximum intensity, as shown in Fig. 3. In a few shots at lower laser intensity, the Cu $K_{\alpha }$ emission is peaked at or slightly after the laser maximum.

### Laser plasma instabilities

In order to investigate the origin of HE, Stimulated Raman Scattering and Two Plasmon Decay were characterized by calorimetry, time-integrated and time-resolved spectroscopy.

**Figure 4:**
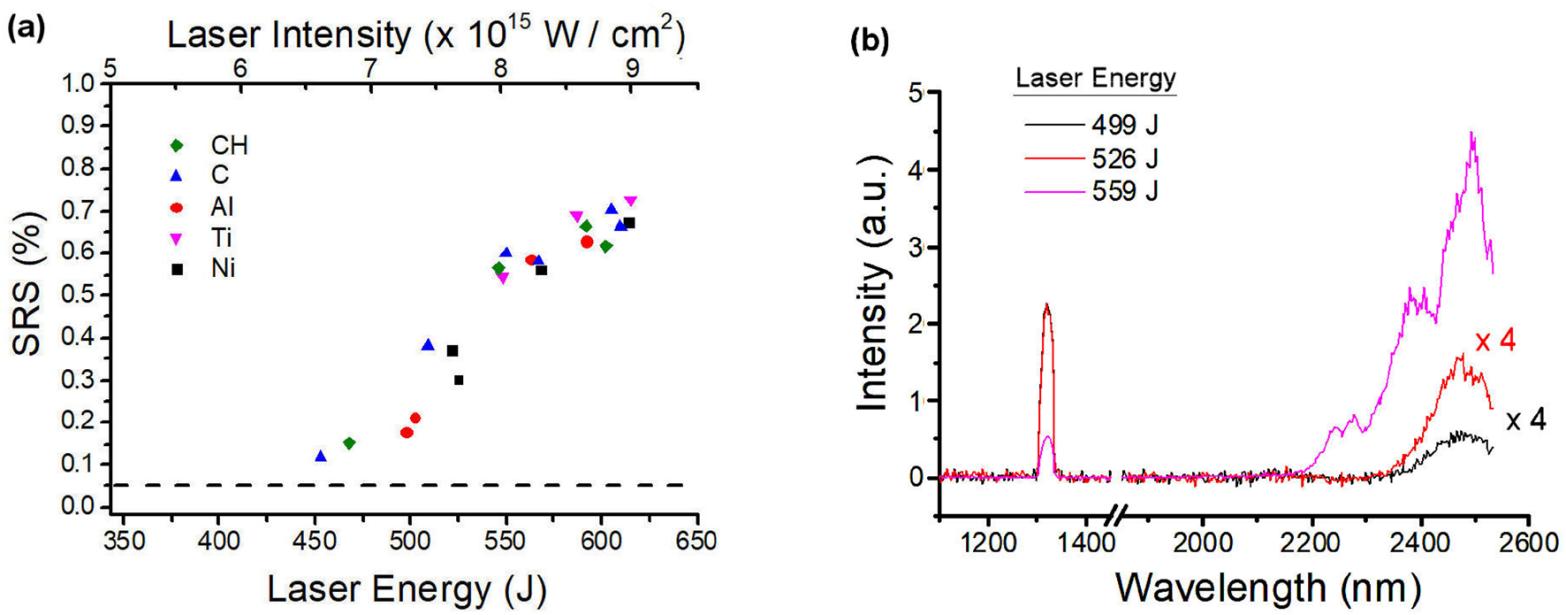
(**a**) values of SRS reflectivity measured by the calorimeter for different targets vs. the laser energy/intensity. Laser intensity is here calculated by considering a laser pulse duration $\tau = 330$ ps. (**b**) SRS spectra obtained at different laser energies and parylene-N ablators.

**Time-integrated spectra** of the infrared scattered light allowed a qualitative insight into the SRS process, showing a signal beginning at $\lambda =$ 2200–2350 nm and extending to the higher wavelength limit of the spectrometer $\lambda =2530$ nm, in agreement with previous experiments^28^. The lower wavelength limit corresponds to the Landau cutoff condition $k_e \lambda _D \approx 0.3$ for plasma temperatures of 3–4 keV ($\lambda =2200$ and $\lambda =2350$ for T = 3 keV and T = 4 keV, respectively); this suggests that Landau damping of plasma waves restricts the SRS growth to regions with electron densities larger than 0.13–0.15 $n_c$. As shown in the IR spectra in Fig. 4, the intensity of the signal increases with laser energy; however, the reproducibility of the SRS intensity at a fixed laser energy is quite low, due to the small plasma region probed by this diagnostics. It is also worth noticing that SRS spectra peaked at $\lambda \approx 2450$ nm imply plasma waves with a phase velocity $v_{ph}\approx 0.38\;c$, which are expected to generate HE with energy $\approx$ 40 keV.

**Calorimetric measurements** show that a large fraction of laser energy, spanning from 10 to 30$\%$, is backscattered in the focussing cone at wavelengths close to the laser frequency; this amount of energy includes both SBS and laser light and does not show any dependence on the laser intensity in the range explored. Since the aim of our work was to understand the origin of HE, the diagnostics were not designed to distinguish the features of SBS.

The energy scattered by SRS light is estimated in the range (0.1–0.8)$\%$, rising with laser intensity (Fig. 4a). Due to the poor transmissivity of the light in the infrared range, the uncertainty on this value is ${\approx }50\%$; these values are slightly lower than those obtained in a previous experimental campaign at PALS with similar energy and focussing conditions, i.e. (0.6–4)$\%$^28^. Shots with different targets also reveal that SRS reflectivity is not affected by the composition of the ablation layer (Fig. 4a). Comparing the SRS backscattered energy, measured by the calorimeter and corrected by the line transmissivity, and the HE energy calculated by Geant4 analysis of BSC data, we obtain a clear correlation (Fig. 3b), suggesting that SRS could be the main source of HE. The discrepancy between the absolute values of SRS and HE energy is explainable by a partial reabsorption of SRS scattered light before exiting the plasma; this probably involves collisional and also non collisional mechanisms. This mechanism was suggested by numerical particle in cell simulations carried out for similar interaction conditions and reported in Ref.^28^, where the obtained SRS reflectivity was ${\approx } 3\%$ while HE energy was ${\approx } 10\%$. The leading role of SRS in HE generation is also suggested by the expected temperature of SRS-driven HE with the values plotted in Fig. 2.

**Time-resolved spectral characterization of** ${{\frac{3}{2}}\omega _0}$ **light** results from the Thomson scattering of light waves (or its harmonics) with plasma waves excited by parametric instabilities.

**Figure 5:**
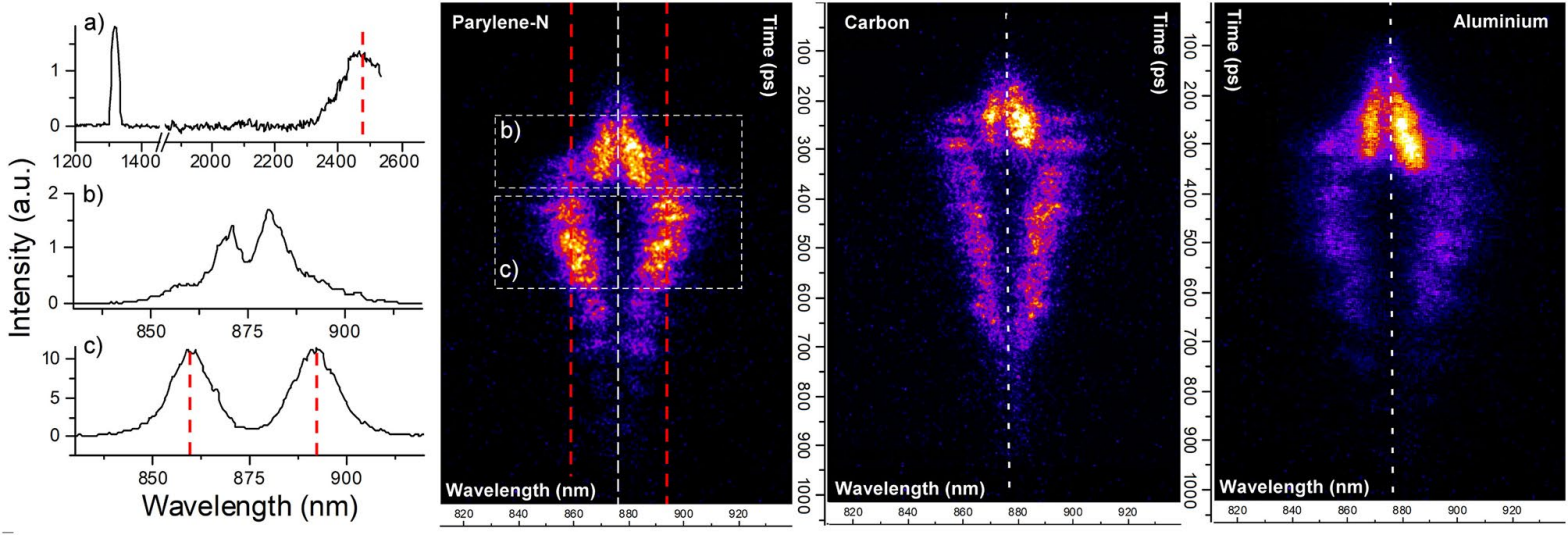
Time- and high-spectral resolved measurement of ${\frac{3}{2}}\omega _0$ light measured in high intensity shots on a parylene-N ($I=7.7\cdot 10^{15}$ W/cm$^2$), carbon ($I=6.8\cdot 10^{15}$ W/cm$^2$) and aluminium ($I=8.7\cdot 10^{15}$ W/cm$^2$) ablators. In the temporal scale, the time is set to zero on the top of the image. The subplot (**a**) shows the time-integrated spectrum obtained by the IR spectrometer for the same shot shown for parylene-N, while subplots (**b**) and (**c**) reports the spectra obtained by time-integrating the signals in the rectangles (**b**) and (**c**) drawn in the streaked image.

**Figure 6:**
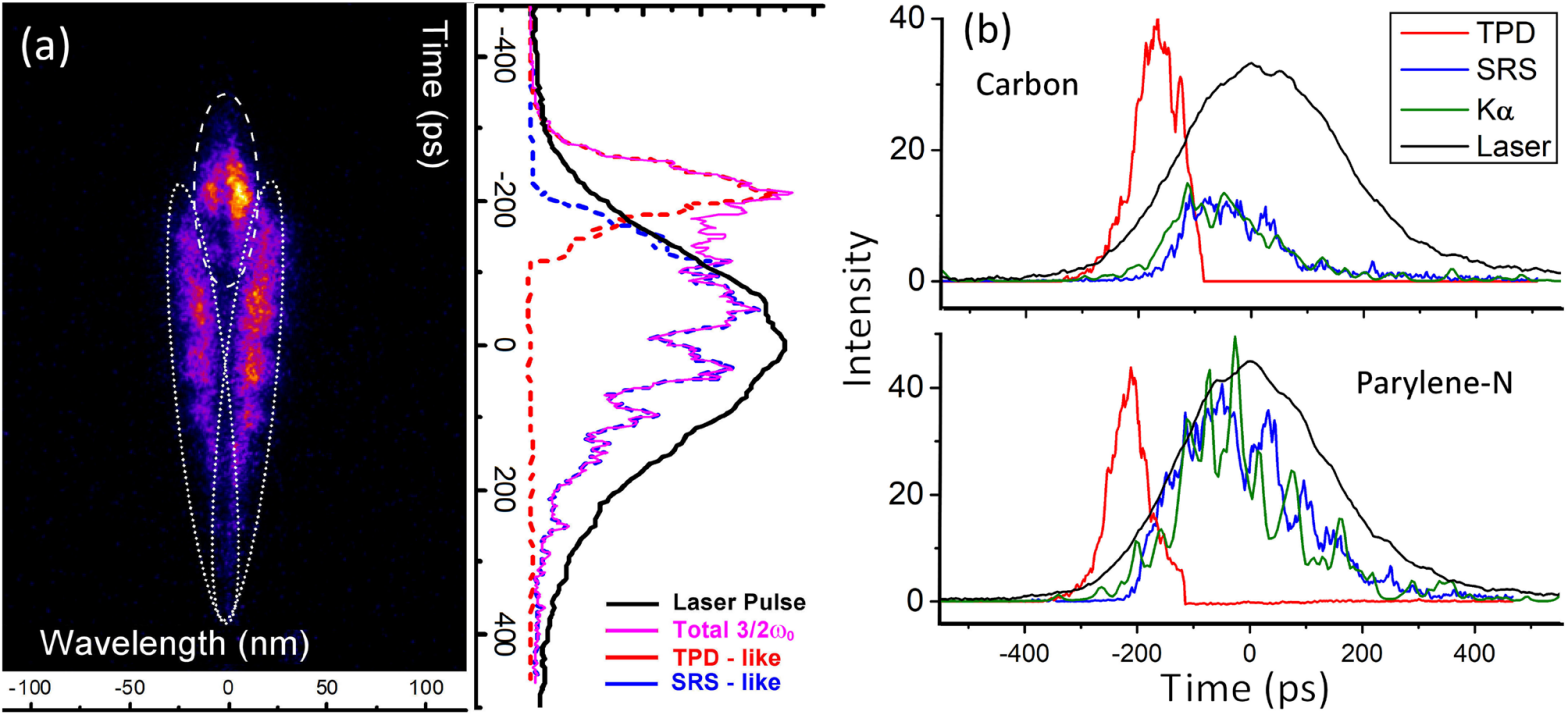
(**a**) Time-resolved measurement of ${\frac{3}{2}}\omega _0$ light induced by a laser pulse at intensity $I=8.2\cdot 10^{15}$ W/cm$^2$ impinging over a parylene-N ablator. On the right, the time profile of spectrally-integrated ${\frac{3}{2}}\omega _0$ signal, and the time profiles of its components produced by TPD (region encircled by dashed line) and by SRS (region encircled by dotted line) are plotted along with the laser pulse profile. (**b**) Temporal profile of TPD and SRS features extracted from ${\frac{3}{2}}\omega _0$ spectra, $K_{\alpha }$ emission and laser pulse for shots over parylene-N (top) and carbon (bottom) multilayer targets. The relative intensity of the different curves is arbitrary. The shot on parylene-N target is the same reported in frame (**a**).

As shown in Fig. 5, the spectra are qualitatively similar for all targets (here only data from parylene-N, carbon and aluminium targets are shown); the signal consists of two spectral components on the red and blue sides of wavelength $\lambda ={\frac{2}{3}}\lambda _0=876$ nm (white dashed line). As previously discussed in Ref.^28^, we suggest that both components include signals derived from SRS- and TPD-driven plasma waves. The disentanglement of the two contributions, and therefore the interpretation of the ${\frac{3}{2}}\omega _0$ spectra, however, is still uncertain and deserves further experimental or theoretical investigation. As shown in the following, some experimental and numerical findings suggest here a partial interpretation of the spectra.

**Figure 7:**
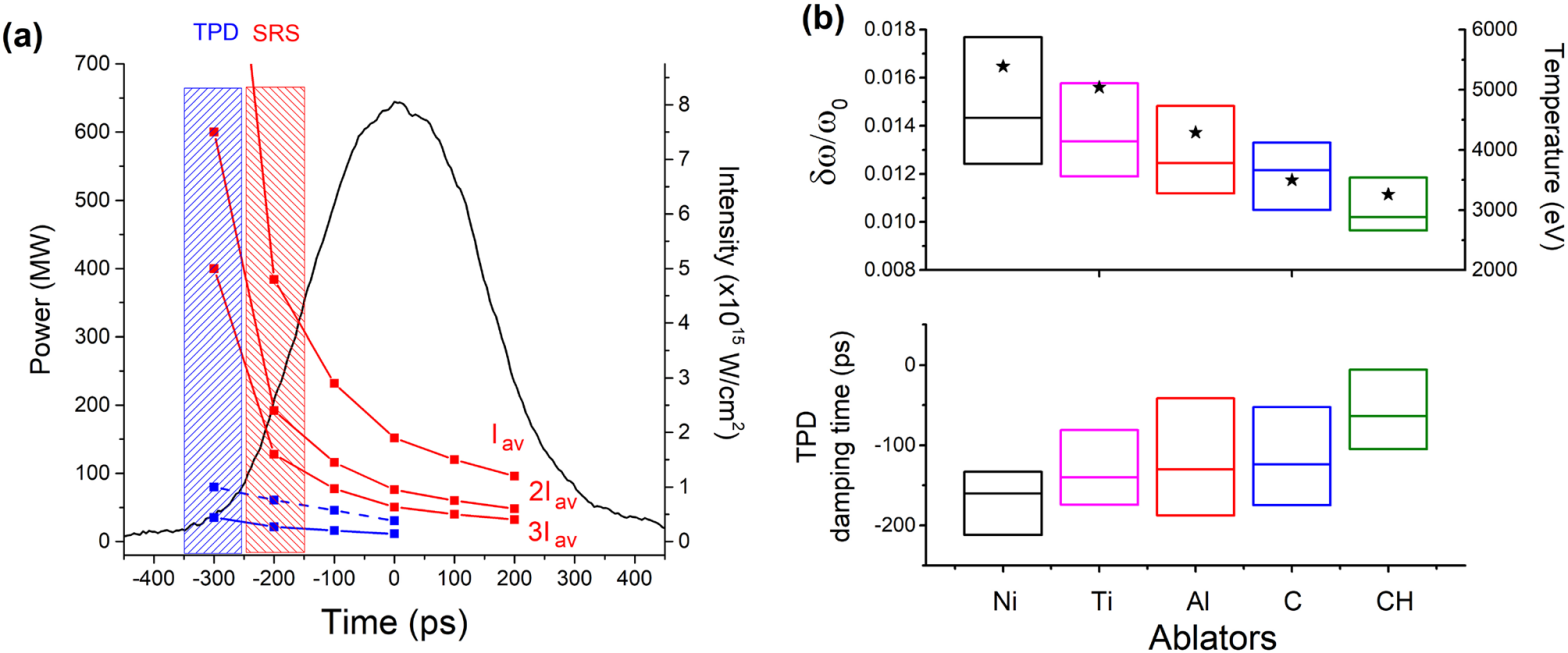
(**a**) Blue and red shaded regions represent the onset time of the two components observed in $\frac{3}{2}$ $\omega _0$ spectra along the laser pulse temporal profile (black line). The solid and dashed blue lines represent the thresholds of absolute and convective TPD (calculated at a density of 0.23 *n*_*c*_). The red lines represent the convective threshold of SRS in speckles with intensity $I=\langle I \rangle , \; 2\langle I \rangle , \; 3\langle I \rangle$. (**b**) Above, frequency splitting $\delta \omega /\omega _0$ and, below, damping time of TPD feature in ${\frac{3}{2}}\omega _0$ spectrum vs. ablator composition (time zero corresponds to the laser peak); the horizontal line is the median of the values. The black stars in the above panel represent the values of plasma temperature at the laser peak time.

Red and blue wings are close to ${\frac{3}{2}}\omega _0$ at the beginning of the interaction and detach with time forming two intense lobes that reach a splitting $\delta \omega \equiv \omega -\frac{3}{2}\omega _0$ in the range of $(0.010-0.016)$ $\omega _0$ (subplot b in Fig. 5), before disappearing. At times comparable to the maximum splitting of these lobes (t = 250–350 ps in the figure), a weaker signal emerges at longer and shorter wavelengths, reaching a maximum splitting $\delta \omega =(0.028-0.044)$ $\omega _0$ (subplot c in Fig. 5) and successively getting closer to 3/2$\omega _0$ at later times of interaction. In order to find the origin of these signals, it is useful to compare their time of emission with respect to the laser peak, that was here obtained by absolute time calibration in low-spectral resolution measurements. A time-resolved spectrum from a parylene-N ablator target, where the time zero corresponds to the laser peak time, is shown in Fig. 6a. The signal of the early two intense lobes (encircled by a dashed line in left panel of Fig. 6a) emerges $\approx$ 350–250 ps before the laser peak when laser intensity is lower than $5\cdot 10^{14}$ W/cm$^2$; successively, this signal reaches a maximum intensity and splitting at times of $-200\pm 50$ ps and finally disappears well before the peak of the laser pulse. In shots with a lower laser intensity $I= (3-4)\cdot 10^{15}$ W/cm$^2$, this signal shifts to later times of interaction, and reaches a maximum around the laser peak. The two lobes with larger frequency shift (encircled by a dotted line in left panel of Fig. 6a) appear $\approx$ 150–200 ps before the laser peak and are well visible up to 200 ps after the laser peak with a weaker tail at longer times. The vertical lineout of the spectrally integrated $3/2\omega _0$ signal and of the two components visible in the regions delimited by dashed and dotted lines in the figure, are plotted versus the laser pulse profile (black curve) in the right panel of Fig. 6b.

#### TPD features in ${\frac{3}{2}}\omega _0$ spectra

The small frequency splitting of the earlier lobes, the asymmetric intensity of red and blue wings and the laser intensity at the time of signal appearance suggest that this signal is produced by Thomson scattering of laser light with TPD-driven plasma waves. The consistency between its experimental onset time (blue shaded area) and the TPD thresholds can be observed in Fig. 7a; here, solid and dashed blue lines represent the time-dependent thresholds of TPD driven in absolute and convective regimes, calculated by considering temperature and density scalelength values given by hydrodynamic simulations for a parylene-N ablator. According to the figure, both thresholds are reached at times of $\approx -300$ ps, overlapping with the experimental onset time of the former component in the ${\frac{3}{2}}\omega _0$ spectrum.

An accurate description of TPD instability can be obtained from the values of frequency splitting. Assuming that the instability is driven along the hyperbola of maximum growth rate, the frequency splitting is determined by the local values of both temperature and electron density, where $\delta \omega /\omega _0\approx 4.4\cdot 10^{-3}\kappa T_{e,keV}$ with $\kappa = \mathbf {k_B}\cdot \mathbf {k_0}/k_0^2 - 1/2$ and $\mathbf {k_B}$,$\mathbf {k_0}$ are the wavevectors of the blue plasma wave driven by TPD and of the laser light, respectively. The measured values of $\delta \omega / \omega _0$ exclude that TPD is here driven as an absolute instability in the proximity of $n_c/4$ region, since this would imply a plasma temperature of $\approx 5$ keV. This is not possible at times well before the laser peak, when CHIC simulations estimate plasma temperatures lower than 2 keV. Conversely, the results suggest that TPD grows with a convective character, reaches its maximum growth at densities $n\approx 0.23$ $n_c$ where Landau damping is weak ($k_e\lambda _D\approx$ 0.21–0.23), and successively is damped and abruptly stops. In the final stage of evolution, the high plasma temperature and the lower densities of TPD excitation make the Landau damping strong ($k_e\lambda _D\approx$ 0.27–0.30). At the time of maximum intensity of the early $3/2\omega _0$ lobes, TPD growth occurs across densities between 0.2 and 0.245 $n_c$.

#### SRS-like features in ${\frac{3}{2}}\omega _0$ spectra

The ${\frac{3}{2}}\omega _0$ signal with larger frequency splitting (dotted region in left panel of Fig. 6a) emerges before the laser peak at times between − 220 ps and − 70 ps and tends to disappear at $\approx 200$ ps after the laser peak. Usually, it coexists with the TPD feature (early lobes) for a time of ${\approx }100$ ps.

As previously discussed in Ref.^28^, this signal can be hardly associated to TPD plasma waves driven on the maximum growth hyperbola; if this is the case, TPD would be driven at densities too low ($n\approx 0.1$ $n_c$) and would be strongly Landau damped ($k_e\lambda _D\approx$ 0.8–1.0). The comparison with the IR spectra suggests that the shift of this signal with respect to the nominal ${\frac{3}{2}}\omega _0$ frequency is consistent with the Thomson scattering of SRS plasma waves; this is clearly shown in Fig. 5a, where the SRS peak in the IR spectrum is highlighted by a red dashed line, and correspondingly a red dashed line is reported in the ${\frac{3}{2}}\omega _0$ spectrum at the Thomson scattering wavelength produced by laser light $\omega _0 + \omega _e^{SRS}$ (and at the symmetric shift on the blue side). By assuming a SRS origin of the red lobe ($\omega _0 + \omega _e^{SRS}$), the maximum intensity corresponds to densities $n\approx (0.19-0.20)$ $n_c$, in agreement with time-integrated IR spectra. The onset time of this signal corresponds to laser intensities in the range (1–5) $\cdot 10^{15}$ W/cm$^2$, which are consistent with the convective SRS threshold $I_{thres}^{SRS}=$ (3.5–5) $\cdot 10^{15}$ W/cm$^2$ calculated by considering density scalelengths $L_n=$ 30–50 $\upmu$m obtained at the relevant times by CHIC hydrodynamic simulations. This can be observed in Fig. 7a, where the time window of the large shifted, SRS-like, lobes (red shaded area) overlaps with the times of intersection between laser intensity and SRS threshold (upper red curve). Figure 7a also shows that the red shaded region extends to lower laser intensities, which can be explained by the onset of SRS in laser speckles with higher local intensity, reaching the thresholds at early times; this is clearly shown by the different red curves in the figure expressing the SRS threshold in speckles with intensity $I_{sp}=I_0,2I_0, 3I_0$. At late times of interaction, the frequency splitting between red and blue wing becomes smaller, suggesting that SRS moves to regions closer to the quarter critical density; in this region, however, the splitting becomes again consistent with a signal produced by TPD plasma waves, making more difficult the disentanglement between SRS and TPD origin.

It is worth remarking that the origin of ${\frac{3}{2}}\omega _0$ spectrum is not completely clear and needs a more detailed investigation. While the red wing signal $\omega _R$ could be in fact produced by the nonlinear coupling between laser (or SBS-scattered) light $\omega _0$ with SRS plasma waves $\omega _e^{SRS}$, the origin of the blue wing signal $\omega _B$ is uncertain. In a previous experimental campaign at PALS carried out in similar interaction conditions^28^, we clearly observed double-peaked spectral features at ${\frac{3}{2}}\omega _0$, ${\frac{5}{2}}\omega _0$ and ${\frac{7}{2}}\omega _0$ frequencies, showing evidence of the non linear coupling of laser harmonics ($2\omega _0$ and $3\omega _0$) with electron plasma waves; there, we already suggested the possibility of Thomson downscattering of $2\omega _0$ harmonic with SRS plasma waves, resulting in a blue-shifted ${\frac{3}{2}}\omega _0$ feature, i.e. $\omega _B=2\omega _0-\omega _e^{SRS}$, as that observed in the present experiment. This explanation, however, would suggest a much lower intensity of the blue wing signal with respect to the red wing one, unless the intensities are finely balanced by the matching conditions of the two processes. This expectation is in contrast with the experimental data shown above. This therefore casts doubts on the interpretation of Thomson downscattering of $2\omega _0$ harmonic as a source of ${\frac{3}{2}}\omega _0$ blue feature.

A further indication that the late-time lobes in ${\frac{3}{2}}\omega _0$ spectra could be associated to SRS is also given by the results shown in Ref.^29^, where 2D kinetic simulations of laser interaction in conditions close to the present experiment, performed with the EPOCH code, are reported. Simulations are performed for both S-polarization and P-polarization laser pulses. The simulated scattered spectra, reported in Fig. 5 of Ref.^29^, show clear ${\frac{3}{2}}\omega _0$ spectra with frequency splitting similar to those measured here; such spectra, however, are visible also in S-polarization simulations, where TPD is not observed (plasma waves driven by TPD propagate in the polarization plane of the laser) and 2$\omega _0$ peak is missing. The authors conclude that ${\frac{3}{2}}\omega _0$ spectra include a significant contribution of SRS. Moreover, the absence of correlation between 3/2$\omega _0$ features and 2$\omega _0$ peaks (strong for P-polarization and lacking in S-polarization simulations) suggests that Thomson downscattering of 2$\omega _0$ light is not responsible for the generation of the blue-shifted ${\frac{3}{2}}\omega _0$ peak; the mechanism generating this signal from SRS-waves, therefore, calls for a further experimental and numerical research.

#### Comparison of ${\frac{3}{2}}\omega _0$ and K$_\alpha$ emission timing

It is interesting now to compare the time-profiles of TPD-like and SRS-like features in ${\frac{3}{2}}\omega _0$ spectra with time-resolved Cu $K_{\alpha }$ emission. Figure 6b shows a substantial temporal overlap of Cu $K_{\alpha }$ with the SRS-like feature. Moreover, the typical duration of Cu $K_{\alpha }$ emission is similar to that of this feature and much longer than that of the TPD-feature ($FWHM=70-120$ ps). This suggests that HE are not driven by TPD, but are associated to the mechanism originating the second lobe, which could be SRS. An additional piece of information is given by the spatial extension of Cu $K_{\alpha }$ emission, which is $FWHM=200-250$ $\upmu$m for most of the shots. By considering the laser spot size ($FWHM=100$ $\upmu$m), the distance travelled into the target ($\approx 70$ $\upmu$m) and the hydrodynamic expansion of the plasma in front of the target, a divergence of HE $\Delta \phi \approx 10^{\circ }$ can be calculated. This neglects the effects of collisions into the target and of magnetic fields in the electron propagation. This value is much lower than the expected divergence of HE produced by TPD in a saturated regime, i.e driven at laser intensity well above the TPD threshold.

#### Effect of the ablator composition

Finally, it is interesting to discuss the effect of plasma temperature on laser-plasma interaction, experimentally tuned by changing the ablator composition. As shown in Figs. 2 and 4, the composition of the interaction layer does not appreciably affect the SRS reflectivity, the SRS spectra nor the temperature of the HE. This observation is in agreement with hydrodynamic simulations, showing that the different atomic number of the ablator does not produce an appreciable difference in the density scalelength of the plasma, affeting the SRS growth rate, because of the short duration of the laser pulse. Moreover, differently from other experiments^30,31^, we do not observe an enhancement of laser-plasma interaction in H-rich targets (e.g. the parylene-N), which is often associated with the saturation of SRS by Langmuir Decay Instability. We believe that the reason is not related to the presence of the Al flash coating in front of the parylene-N layer, since the aluminium plasma is expected to be already at densities lower than 0.15 $n_c$ at times before the laser peak.

The overall features of the ${\frac{3}{2}}\omega _0$ spectra described above were observed for all the targets. However, the timing of the earlier and later signals, which we associate to TPD and SRS, shows a dependence on the ablator. As discussed above, TPD begins to grow at a comparable time for all the targets, that is consistent with the TPD thresholds given by linear theory^15,20^. However, the frequency splitting of the TPD lobes increases with the atomic mass of the ablator, going from $\delta \omega / \omega _0 =$ (0.93–1.17) $\cdot 10^{-2}$ for parylene-N to $\delta \omega / \omega _0 =$ (1.23–1.46) $\cdot 10^{-2}$ for nickel targets (Fig. 7b, top). Considering the hydrodynamic simulations, this variation can be explained by the higher plasma temperatures obtained for high-Z ablators, rather than by a different density where TPD is driven. The time of TPD damping is also markedly affected by the ablator composition, where the instability is damped at earlier times in high-Z ablators, i.e. $t\approx -150$ ps for Ni, and at later times for low-Z ablators, i.e. $t\approx -50$ ps for parylene-N (Fig. 7b, bottom). Since the temperature of the plasma rises with the atomic number of the ablator, the earlier disappearance of the TPD for higher Z ablators could be related to the stronger Landau damping of plasma waves, when their excitation has moved to lower density regions ($\approx$ 0.20–0.21 $n_c$); however, the complete cutoff of TPD, even at densities closer to $n_c/4$, can be hardly explained by this mechanism.

Besides, the onset of the SRS-like feature in the ${\frac{3}{2}}\omega _0$ spectra is observed at earlier times for high-Z ablators, i.e. between − 250 ps and − 300 ps for Ni vs. a time between − 200 ps and − 70 ps for parylene-N. Classical theory of SRS, however, can not explain this observation, since the threshold of convective SRS is not directly affected by plasma temperature. What is more, since the density where SRS is driven is comparable for all the targets, we would expect that Landau damping of electron plasma waves driven by SRS to be larger for high-Z ablators, therefore enhancing the SRS threshold. This is opposite to the time of appearance of the SRS-like signal.

Precious information for interpreting these data are given in Ref.^28^, reporting a picture of laser-plasma interaction in the same experimental conditions of this experiment, as obtained by 2D Particle In Cell simulations at laser peak time. Simulations showed that the interaction is dominated by a strong filamentation and SBS driven into filaments, reflecting large part of laser energy; moreover, while SRS was clearly observed, TPD was immediately damped, in agreement with the absence of TPD signatures in experiments at the laser peak time. A more detailed picture, in same conditions, was reported in Ref.^29^ where Particle In Cell simulations in 2D and 3D geometry were compared and critically discussed. Simulations confirmed the importance of filamentation of speckles, drilling a channel of low density, where parametric instabilities are suppressed for the strong Landau damping of the electron plasma waves; TPD and SRS, however, marginally grow at the edges and at the head of the channel, but with a reduced growth rate for the steepening of the density profile and for the pump depletion produced by SBS.

This picture suggests that TPD disappearance here observed could be related to the formation of laser filaments and/or by the strong pump depletion due to SBS driven at lower densities. In this case, the density steeping at the edges of the filament could produce the TPD cutoff, enhancing the TPD threshold, while the dependence on the atomic number Z of the ablator could be produced by the dependence of the TPD threshold on the plasma temperature or on the Landau damping of the plasma waves. The dependence on Z could be also produced by the formation of filaments and laser beam spray, produced by Forward Stimulated Brillouin Scattering (FSBS) in beam speckles^32^; in that case, the dependence on Z descends from the excitation of ion acoustic fluctuations by thermal effects^33^.

The onset of filamentation, could also drive the ignition of SRS for the enhancement of laser local intensities; however, it remains uncertain why SRS should be less affected by density steepening at later times of channel formation and continue to grow at the edges of the filaments for the remaining part of the interaction. The time overlap between the SRS onset and the TPD growth at low densities in the saturated regime could also suggest that these two processes are in some way correlated. SRS is in fact usually driven at densities $n\approx 0.20\; n_c$ and at times when TPD has moved to similar densities. It is therefore possible that ion density perturbation, Langmuir turbulence, or even HE bursts driven by TPD in saturated regime produce a non linear coupling between TPD and SRS^34^ growth, and could act as a seed for the ignition of SRS backscattering.

## Conclusions

The experiment described in this paper was conceived with two different aims, (a) understanding the origin of HE and (b) investigating the dependence of SRS/TPD on the plasma temperature, at laser intensities relevant for the Shock Ignition scheme to Inertial Confinement Fusion. The correlation of HE energy and SRS reflectivity and the low divergence of HE suggest that SRS is the main source of HE, despite TPD being visible in light scattered spectra. Furthermore the temperature estimated for HE is in agreement with the value obtained by considering the phase velocity of plasma waves driven by SRS, as obtained by plasma emission spectroscopy. This origin of HE is reinforced by comparing the timing of emission peaks in the ${\frac{3}{2}}\omega _0$ spectra, with the timing of the Cu $K_{\alpha }$ emission, which is a tracer of HE generation. However, a more detailed investigation of the mechanisms producing the different features visible in the ${\frac{3}{2}}\omega _0$ is needed, in order to make this diagnostics unambiguous and more reliable; in particular, the mechanism producing the highly shifted peaks in ${\frac{3}{2}}\omega _0$ emission, which we associate to SRS, is in fact uncertain.

Valuable information on the dependence of SRS and TPD on the plasma temperature could be obtained by comparing measurements with different ablators. These are expected to produce plasmas with maximum temperatures in the range 3–5.4 keV. Calorimetric data suggest that SRS total reflectivity is not affected by the plasma temperature or by the atomic number Z of the ablator. However, ${\frac{3}{2}}\omega _0$ spectra show that TPD is damped at earlier times in hotter plasmas, and this is accompanied by an earlier appearance of the feature in the ${\frac{3}{2}}\omega _0$ spectrum that we associated to SRS. The transition from a TPD-dominated to a SRS-dominated regime could be here produced by the onset of filamentation, significantly modifying the density profile in the plasma, and the dependence on Z could be explained by the dependence of TPD threshold on the plasma temperature and on the Landau damping. In conclusion, similarly to experiments carried out at Vulcan, LMJ and Omega facilities in conditions of longer density scalelength but lower plasma temperature, the present experiment suggests that SRS is the predominant parametric instability at Shock Ignition intensities, responsible for the generation of the majority of the hot electrons, while TPD is damped by the concomitant effect of filamentation and multi-keV plasma temperature, or by pump depletion of laser light.

## Data Availability

The datasets used and/or analysed during the current study are available from the corresponding author on reasonable request.
